# Conjecture and hypothesis: The importance of reality checks

**DOI:** 10.3762/bjoc.13.60

**Published:** 2017-03-28

**Authors:** David Deamer

**Affiliations:** 1 Department of Biomolecular Engineering, University of California, Santa Cruz CA 95060, USA

**Keywords:** hydrothermal fields, hydrothermal vents, origin of life, polymerization by condensation, protocells

## Abstract

In origins of life research, it is important to understand the difference between conjecture and hypothesis. This commentary explores the difference and recommends alternative hypotheses as a way to advance our understanding of how life can begin on the Earth and other habitable planets. As an example of how this approach can be used, two conditions have been proposed for sites conducive to the origin of life: hydrothermal vents in salty seawater, and fresh water hydrothermal fields associated with volcanic landmasses. These are considered as alternative hypotheses and the accumulating weight of evidence for each site is described and analyzed.

## Introduction

The word conjecture is defined as an opinion based on incomplete information. The word can be taken to be slightly pejorative, but given that conjecture also involves imagination and creative effort, I will argue here that in scientific research there is a natural progression from conjecture to hypothesis to consensus. Conjecture is an idea, hypothesis is a conjecture that can be tested by experiment or observation, and consensus emerges when other interested colleagues agree that evidence supports a hypothesis that has explanatory value. This approach is clearly relevant to origins of life research which is still at a stage where multiple conjectures abound yet vast gaps in knowledge and understanding remain, mostly due to lack of significant funding for research in this area. The result is that only a few dozen laboratories are supported in the global scientific community, in contrast to thousands of scientists investigating health related research or chemistry and physics having applications in industry. Another reason is that the origin of life is best understood in interdisciplinary terms involving knowledge of astronomy, planetary science, biophysics, chemistry and biochemistry, molecular biology and evolution. Relatively few scientists have a taste for research that demands such broad knowledge to make significant advances. The historical development of origins research has been well described by Iris Fry [1] and Antonio Lazcano [2].

## Discussion

Most scientists agree that hypothesis testing is an essential feature of research, and a typical proposal to a funding agency usually has a clearly stated hypothesis. However, there is a very human tendency for investigators to prefer positive results that support their idea. Karl Popper [3] had some good advice in this regard: Don't try to prove an idea is right. Instead, try to falsify it. Those rare ideas that cannot be falsified then emerge from the majority of ideas that fail the testing process. Günther Wächtershäuser [4] recently commented on how Popper's advice can be applied in origins of life research.

Hypothesis testing is an essential feature of good research, but its value can be increased by one additional step which was first clearly stated in 1964 by John Platt [5]. The title of Platt's article was *Strong Inference*, which he defines in the following way:

“Strong inference consists of applying the following steps to every problem in science, formally and explicitly and regularly.

1. Devising alternative hypotheses.
2. Devising crucial experiments ... with alternative possible outcomes, each of which will, as nearly as possible, exclude one or more of the hypotheses.
3. Carrying out the experiment so as to get a clean result.”

Research approaches that incorporate alternative hypotheses avoid the tendency to prefer positive results, because both positive and negative results have value in inferring which of the two alternatives is better supported by accumulating evidence. The aim of this commentary is to describe how alternative hypotheses can be applied to understanding the origin of life, with the focus on a simple question: Did life begin in salty water in a marine environment, or did life begin in fresh water in a terrestrial setting? Although the question seems simple, there are significant ramifications of possible answers for life detection missions to other planetary objects in the solar system.

We can begin with two conjectures and then attempt to turn them into alternative hypotheses. The first conjecture follows from the discovery of hydrothermal vents and observations related to their properties:

- All life requires liquid water
- Most of the water on Earth is in the ocean.
- Hydrothermal vents emerging from the ocean floor are sources of chemical energy.
- Populations of chemotrophic microbial life thrive in hydrothermal vents.

*Conjecture:* life originated in hydrothermal vents and later adapted to fresh water on volcanic and continental land masses. In the absence of alternatives this idea has been accepted as a reasonable suggestion.

Is there an alternative? Here is another list of facts:

- A small fraction of the Earth's water is distilled from seawater and precipitates as fresh water on volcanic land masses.
- The water accumulates in hydrothermal fields that undergo cycles of evaporation and refilling.
- During evaporation, dilute solutes in the water become concentrated films on mineral surfaces.
- If the solutes can undergo chemical or physical interactions, they will do so in the concentrated films.
- The products will accumulate in the pools when water returns either in the form of precipitation or as fluctuations in water levels related to hot springs or geyser activity.

*Conjecture:* life originated in fresh water hydrothermal fields associated with volcanic land masses, then adapted to the salty seawater of the early ocean.

### The current paradigm: Life began in the ocean in salty seawater

Now we can provide a few more details about two geophysical conditions that have been proposed as alternative sites conducive for the origin of life. Hydrothermal vents were discovered in 1977 [6] and were soon proposed to be a likely site for life to begin [7–10]. Hydrothermal vents referred to as black smokers are produced when seawater comes into contact with rocks heated by magma underlying mid-ocean ridges. The hot water dissolves mineral components of the rock and then emerges through the ocean floor where the mineral solutes come out of solution to form characteristic chimneys that emit a black smoke of precipitated metal sulfide particles.

A second type of hydrothermal vent was discovered in 2001 [11] that does not depend on volcanism. Instead they form when seawater reacts with mineral components of peridotite in the sea floor, a process called serpentinization. The reaction produces hydrogen and a strongly alkaline (pH 9–11) hot medium saturated with carbonate. When the warm fluid contacts cooler seawater, calcium carbonate and other minerals precipitate to form white chimney structures.

The hydrogen gas dissolved in the alkaline vent fluid is a potential source of reducing power. Certain microorganisms already use hydrogen for this purpose, so the hydrothermal vent hypothesis proposes that on the prebiotic Earth hydrogen could potentially reduce carbon dioxide to organic compounds that are then incorporated into a primitive metabolism [12]. Lane and Martin [13] noted that the alkaline vent minerals have a porous structure that could serve as cellular compartments with mineral membranes as boundaries. The assumption that the membranes may separate a strongly alkaline medium from mildly acidic Hadean sea water across suggested that a primitive version of chemiosmotic energy transduction might be possible to supply chemical energy for primitive forms of life. Weiss et al. [14] used genomic analysis of vent microorganisms to test the possibility that the last universal common ancestor (LUCA) may have originated in hydrothermal vents.

The iron-sulfur chemistry proposed for hydrothermal vents was tested by Huber and Wächtershäuser [15–16] who simulated vent conditions with boiling mixtures of iron and nickel sulfides to which various reactants were added. They reported that acetic acid, amino acids and peptide bonds could be synthesized under these conditions, and claimed that “The results support the theory of a chemoautotrophic origin of life with a CO-driven, (Fe,Ni)S-dependent primordial metabolism.”

More recently Herschy et al. [17] simulated hydrothermal vent conditions by injecting a solution of potassium phosphate, sodium silicate and sodium sulfide (pH 11) into a second solution of ferrous chloride, sodium bicarbonate and nickel chloride (pH 5). The aim was to determine whether carbon dioxide (present as 10 mM sodium bicarbonate) can be reduced under these conditions, and they were able to detect ≈50 μM formic acid. In a similar laboratory simulation of an alkaline hydrothermal vent, Burcar et al. [18] used mass spectrometry to detect a small yield of dimers produced from adenosine monophosphate circulating in the medium.

### An alternative hypothesis: Life began in terrestrial fresh water

Although most of the Earth's water today is salty seawater, a small fraction (~1%) is present in the form of fresh water distilled by evaporation from the ocean and falling on continental land masses as precipitation. The Hadean Earth did not have continents but was likely to have volcanoes similar to those from the same era still visible on Mars. The volcanism associated with such islands suggests an alternative hydrothermal site we will refer to as hydrothermal fields. Iceland is an analogous site on today's Earth, with several active volcanoes and associated hydrothermal areas supplied by precipitation and dominated by hot springs and geyser activity. In contrast to the single rock-water interface of hydrothermal vents, hydrothermal fields have a more complex array of three interfaces in which minerals, water and atmosphere undergo continuous fluctuations of wetting and drying.

The fluctuating hydrothermal field hypothesis has been used as a model for polymerization reactions in which monomers like amino acids and mononucleotides form peptide and ester bonds of biologically relevant polymers. The idea that evaporation and heat can drive polymerization is obvious and was first proposed years ago [19]. Lahav and White [20] adopted the approach and demonstrated that peptide bonds could be produced using clay as a catalyst. The approach was largely abandoned with the advent of the RNA World scenario that suggested a way for life to begin in solution, rather than by evaporation to dryness. However, polymerization in an aqueous medium requires chemical activation of the monomers, and so far there is no obvious mechanism by which activation can occur. Recent studies have returned to evaporation as a way to drive polymerization reactions [21–22].

There are several advantages to using evaporation in this regard. First, simply concentrating potential reactants adds significant free energy to a system that can be used to drive condensation reactions [23]. Furthermore, if amphiphilic compounds are present they can organize and concentrate reactants within a two dimensional plane with the result that polymerization is enhanced [24–25].

The hydrothermal field hypothesis has been tested in laboratory simulations. For instance, peptide bonds have been produced [26–27] and cycles of drying and rehydration have been shown to drive polymerization of mononucleotides [22,28–29]. Because the resulting polymers can be encapsulated in lipid vesicles, it has been proposed that the resulting protocells are candidates for combinatorial selection and the first steps of evolution [30].

## Conclusion

From the above discussion, alternative conjectures have been published and are available for critical analysis and commentary. How can we turn the two conjectures into John Platt's alternative hypotheses? The answer is simple. We follow Platt's advice to devise critical experiments that will add weight of evidence to either or both of the alternative conjectures which then become testable hypotheses. Here is a proposed list of conditions that seem to be essential prerequisites if cellular life is to originate in one of the two alternative conditions:

- There must be a source of organic compounds relevant to biological processes, such as amino acids, nucleobases, simple sugars and phosphate.
- The organic solutes are likely to be present as very dilute solutions, so there should be a process by which they can be sufficiently concentrated to undergo chemical reactions relevant to cellular life.
- Energy sources must be present in the environment to drive a primitive metabolism and polymerization.
- Products of reactions should accumulate within the site rather than dispersing into the bulk phase environment.
- Biologically relevant polymers are synthesized with chain lengths sufficient to act as catalysts or incorporate genetic information.
- If amphiphilic compounds are present in the mixture, the conditions will allow them to assemble into membranous compartments.
- A plausible physical mechanism can produce encapsulated polymers in the form of protocells and subject them to combinatorial selection.

These conditions can also be considered to be predictions, because each condition in the above list can be tested by observation, by theoretical analysis or in laboratory simulations. If any one of the predictions fails experimentally or is shown to be impossible, for instance by being inconsistent with thermodynamic principles, that alternative can be considered to be falsified. As evidence accumulates, we will be able to judge the relative plausibility and explanatory power of the competing ideas. Continued testing of the alternative hypotheses is essential, because neither has yet reached the level of consensus. In both cases, laboratory simulations will ideally be extended to a second important step, which is to visit the alternative sites and demonstrate that what happens in the laboratory can also occur in the actual conditions of hydrothermal vents or fields.
